# Phenolic Profile and Bioactive Potential of Stems and Seed Kernels of Sweet Cherry Fruit

**DOI:** 10.3390/antiox9121295

**Published:** 2020-12-17

**Authors:** Sílvia Afonso, Ivo Vaz Oliveira, Anne S. Meyer, Alfredo Aires, Maria José Saavedra, Berta Gonçalves

**Affiliations:** 1 Centre for the Research and Technology of Agro-Environmental and Biological Sciences—CITAB, University of Trás-os-Montes e Alto Douro, UTAD, Quinta de Prados, 5000-801 Vila Real, Portugal; ivo.vaz.oliveira@utad.pt (I.V.O.); alfredoa@utad.pt (A.A.); saavedra@utad.pt (M.J.S.); bertag@utad.pt (B.G.); 2 Department of Biotechnology and Biomedicine, Technical University of Denmark, DTU Building 221, DK-2800 Kgs. Lyngby, Denmark; asme@dtu.dk

**Keywords:** prunus avium, HPLC, by-products, antioxidant, antimicrobial

## Abstract

Every year, large quantities of stems and pits are generated during sweet cherry processing, without any substantial use. Although stems are widely recognized by traditional medicine, detailed and feasible information about their bioactive composition or biological value is still scarce, as well as the characterization of kernels. Therefore, we conducted a study in which bioactivity potential of extracts from stems and kernels of four sweet cherry cultivars (*Early Bigi* (grown under net cover (C) and without net cover (NC)), *Burlat*, *Lapins,* and *Van*) were examined. The assays included antioxidant (by 2,2′-azino-bis(3-ethylbenzothiazoline-6-sulfonic) acid (ABTS), 2,2-diphenyl-1-picrylhydrazyl (DPPH) and β-carotene-linoleic acid bleaching assays), and antibacterial activities against important Gram negative and Gram positive bacterial human isolates. Profile and individual phenolic composition of each extract were determined by High-performance liquid chromatography (HPLC) analysis. Extracts from stems of cv. *Lapins* and kernels of *Early Bigi* NC presented high levels of total phenolics, flavonoids, *ortho*-diphenols and saponins. Excepting for cv. *Early Bigi* NC, major phenolic compounds identified in stems and kernels were sakuranetin and catechin, respectively. In cv. *Early Bigi* NC the most abundant compounds were ellagic acid for stems and protocatechuic acid for kernels. In all extracts, antioxidant activities showed a positive correlation with the increments in phenolic compounds. Antimicrobial activity assays showed that only stem’s extracts were capable of inhibiting the growth of Gram positive isolates. This new data is intended to provide new possibilities of valorization of these by-products and their valuable properties.

## 1. Introduction

Sweet cherry is one of the fresh fruits most appreciated by consumers in the temperate areas of Europe [1,2], not only due to its organoleptic characteristics, such as color, brightness, flavor, aroma and texture, but also for consumers’ awareness of its benefits for human health [3,4,5]. Rich in vitamins and minerals, cherries contains different phytochemicals that are closely related to its antioxidant power [3]. This fruit has been recognized as having beneficial medicinal properties such as anticancer, dietary, antioxidant and anti-inflammatory, due to its content of antioxidant compounds [3,5]. The world cherry production has increased from 2.15 to 2.54 million tonnes from 2009-2018, with Turkey, the USA, and Uzbekistan as the main producers, responsible for 50% of the total world production, while Chile, Hong Kong, and the USA are the main exporters [6]. Portugal supplies some of the first cherries in Europe, and has increased its production by about 46% (2009–2018), with recent data referring to more than 17,500 tons of cherries annually produced [6]. Although sweet cherry is mainly commercialized as fresh fruit, a considerable quantity is used after processing as jam, jelly or juice that generates large amounts of by-products, namely stems and pits [7]. There is no substantial use of this waste, which increase environmental and managements costs each year to deal with the excess of such residues. Therefore, a solution to achieve valorization of the excess of this material is urgent [8].

**Figure 1:**
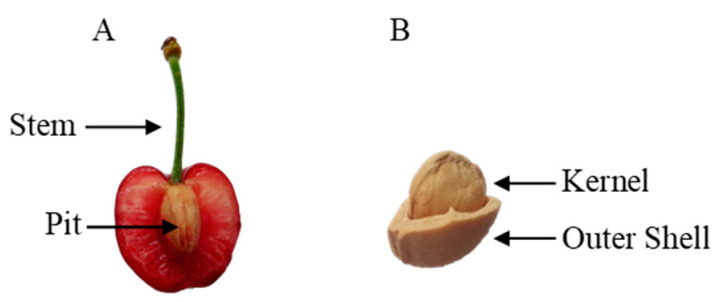
Unvalued sweet cherry by-products: view of sweet cherry stem and pit (**A**) and view of sweet cherry pit (**B**).

Although cherry stems are known by traditional medicine and widely used in infusions and decoctions, due to their claimed sedative, diuretic, and anti-inflammatory properties [9], their study, as well as the characterization of pits, commonly used for therapeutic pillows, only recently have received attention. Various hydroxycinnamic acids, such as *ρ*-cumaric, ferulic, caffeic, chlorogenic and neochlorogenic acids, were reported in stems of *P. avium* [9,10], and important anthocyanins were found in kernels [11], whose oil is rich in lipophilic compounds, such as tocopherols and carotenoids [12] (Figure 1). In this context, the objective of the current work is to evaluate the biological potential of such less used residues, providing a detailed study on their chemical composition, antioxidant, and antimicrobial activities.

## 2. Materials and Methods

### 2.1. Plant Material

Samples were collected from a 6-year old cherry orchard, planted at low elevation, located in Alufinha, S. João de Fontoura, municipality of Resende, Portugal (latitude 41°06′ N, longitude 7°54′ W, altitude 140 m). Fruit from four cherry cultivars, in the commercial ripeness stage, were selected for testing: *Early Bigi* and *Burlat* (cultivars of early maturity), *Van* (cultivar of intermediate maturity) and *Lapins* (cultivar of late maturity). Cultivar *Early Bigi* was grown in two different modes: using net cover over the trees (C) and without net cover (NC). After selecting ten homogeneous trees, three replicates of about 2 kg of cherry per cultivar were collected (May 2019). Stems and pits were removed, with kernels removed from the pits, and both stems and kernels were freeze-dried and then ground to a fine powder used in all analysis.

### 2.2. Chemical Composition and Antioxidant Extracts

#### 2.2.1. Extraction Procedure

For chemical composition and antioxidant activities assays, extracts were prepared by mixing 40 mg dry weight (DW) of samples of each sweet cherry cultivar with 1 mL of 70% methanol, thoroughly in a vortex. The mixtures were heated at 70 °C during 30 min, and then centrifuged at 13,000 rpm, 1 °C for 15 min (Eppendorf Centrifuge 5804 R, Hamburg, Germany). The supernatants were collected and filtered with Spartan filters (0.2 µm) to HPLC amber vials.

#### 2.2.2. Total Phenolics and Flavonoids

The methodology of Singleton and Rossi [13] was used for the quantification of total phenolics, with minor modifications: 20 µL of extract were added with 100 µL of Folin Ciocalteu’s phenol reagent (1:10 in bidistilled H_2_O) and 80 µL of 7.5% Na_2_CO_3_ in a 96-well microplate (Multiskan™ FC Microplate Photometer, Waltham, MA, USA). The microplate was incubated for 15 min at 45 °C, in the dark. Afterward, the absorbance values against a blank were recorded at 765 nm in a microplate reader (Multiskan GO Microplate Spectrophotometer, Thermo Scientific, Vantaa, Finland). A standard curve with gallic acid at different concentrations was performed and total phenolics results were expressed as mg gallic acid equivalent (GAE)/g DW as the mean ± standard deviation (SD) of three replicates.

The total flavonoid content was determined using the colorimetric method described in Dewanto et al. [14], with some modification. In a 96-well microplate, 25 µL of extract was mixed with 100 µL of ultra-pure water, and 10 µL of 5% NaNO_2_. The microplate was then placed in the dark at room temperature. After 5 min, 15 µL of 10% AlCl_3_ was added and the microplate incubated again at room temperature in the dark for 6 min. Then, 50 µL of NaOH 1M, and 50 µL of ultra-pure water were added. The absorbance values were measured against a blank at 510 nm. A standard curve with catechin at different concentrations was performed to quantify total flavonoid content, which values were expressed as mg catechin equivalent (CE)/g DW as the mean ± standard deviation (SD) of three replicates.

#### 2.2.3. Quantification of Ortho-Diphenols

The method used for the determination of *ortho*-diphenols was adapted from Garcia et al. [15]. First, 20 µL of extract were mixed with 100 µL of ultra-pure water. Then 80 µL of phosphate buffer (pH 6.5, 0.1 M) was added, followed by 160 µL of 5% sodium molibdate (Na_2_MoO_4_·2H_2_O) solution. The microplate was left to stand in the dark for 15 min and the absorbance was measured at 370 nm against a blank reagent. Caffeic acid was used as standard to prepare a calibration curve and *ortho*-diphenolic content was expressed as caffeic acid equivalents per g of sample (mg CAE/g DW) as the mean ± standard deviation (SD) of three replicates.

#### 2.2.4. Saponins

For saponin quantification, extractions were performed as referred by Chen et al. [16], with modifications. Briefly, 200 mg of plant material was extracted with 5 mL of 95% (*v*/*v*) ethanol and sonicated for 1 h at room temperature. After extraction, the contents were filtered and stored in amber vials. In 96-well plate, 43 µL of sample was mixed with 34 µL perchloric acid and 9 µL of 5% vanillic acid. Plates were incubated at 60 °C for 20 min, and 214 µL of glacial acetic acid were added. Absorbances were read at 554 nm, and results are expressed using a calibration curve of diosgenin, as diosgenin equivalents per g of sample (mg DE/g DW) as the mean ± standard deviation (SD) of three replicates.

#### 2.2.5. Determination of Chlorophyll a, b and Total Carotenoids

The determination of pigments (only for stems) was performed according to an adaptation of the methods from Lichtentaler and Wellburnn [17], Warren [18] and Pompelli et al. [19]. Briefly, 100 mg of each stems’ samples was extracted with 5 mL of 80% acetone with 0.01% of CaCO_3_, homogenized and centrifuged at 4000 rpm, 20 °C, during 5 min. The absorbance of 200 µL of each sample was recorded at 470, 645 and 662 wavelengths against a blank (pure solvent used in pigments extraction instead of sample). The concentration of each pigment is calculated from 1 cm corrected pathlength using the following formulas:

Chlorophyll a (µg/mL): Chl a = (11.75 × Abs662) − (2.350 × Abs645)

Chlorophyll b (µg/mL): Chl b = (18.61 × Abs645) − (3.960 × Abs662)

Total Carotenoids (µg/mL): ((1000 × Abs470) − (2.270 × Chl a) − (81.4 × Chl b))/227

#### 2.2.6. Antioxidant Activity

##### ABTS Method

The 2,2′-azino-bis(3-ethylbenzothiazoline-6-sulfonic) acid (ABTS) radical scavenging activity was evaluated in a 96-well microplate using the method of Re et al. [20]. An ABTS radical solution was prepared by mixing 7 mM of ABTS at pH 7.4 (5 mM NaH_2_PO_4_, 5 mM Na_2_HPO_4_, and 154 mM NaCl) with 2.5 mM potassium persulfate, and storing the mixture in the dark at room temperature for 16 h. The mixture was then diluted with ethanol to give an absorbance of 0.70 ± 0.02 units at 734 nm. In each microplate well, 15 µL of extract was mixed with 285 µL of the freshly prepared ABTS solution, and incubated at room temperature in the dark for 10 min. Absorbance values were measured at 734 nm, with ABTS radical scavenging activity expressed from a linear calibration curve of Trolox as µg Trolox equivalent/g DW as the mean ± standard deviation (SD) of three replicates.

##### DPPH Method

The 2,2-diphenyl-1-picrylhydrazyl (DPPH) antioxidant activity assay was performed by spectrophotometry as described by Siddhraju and Becker [21]: in a 96-well microplate, 15 μL of extract was combined with 285 μL of freshly prepared methanolic radical DPPH solution (60 µM). The microplates were covered with foil paper and left for 30 min at room temperature. The reduction in absorbance was measured at 517 nm. DPPH was expressed from the linear calibration curve of Trolox as µg Trolox equivalent/g DW as the mean ± standard deviation (SD) of three replicates.

##### FRAP Method

The Ferric Reducing Activity Power (FRAP) assay was performed as described by Stratil et al. [22]. Briefly, a volume of 10 mM solution of 2,4,6-Tri(2-pyridyl)-S-triazine (TPTZ) in 40 mM HCl was mixed with the same volume of 20 mM FeCl_3_·6H_2_O and 10 times volume of acetate buffer pH 3.6 (3.1 g of sodium acetate and 16 mL of glacial acetic acid 100% per L). Afterwards, 275 µL of the Fe^3+^-TPTZ mixture was combined with 25 µL extract, and incubated. After 5 min, the absorbances were recorded at 593 nm, and FRAP was expressed from the linear calibration curve of Trolox as µg Trolox equivalent/g DW as the mean ± standard deviation (SD) of three replicates.

##### β-Carotene-Linoleic Acid Bleaching Assay

The β-carotene-linoleic acid bleaching assay described by Salleh et al. [23] was used, with minor modifications. A mixture of β-carotene and linoleic acid was prepared by adding 0.5 mg β-carotene with 1 mL chloroform (HPLC grade), 25 µL linoleic acid and 200 mg Tween 40. After complete evaporation of the chloroform under vacuum at 40 °C, 100 mL of distilled water was added to the residue, which was gently mixed to form a yellowish emulsion. Then, 50 µL of extract was combined with 0.25 µL of the yellowish emulsion and mixed thoroughly. The mixture was then incubated in a water bath at 50 °C for 2 h, followed by the measurement of absorbance values at 470 nm against a blank. Percentage inhibitions (I%) of the extracts were calculated using the following equation:

I% = (A_β-carotene after 2 h_/A_initial β-carotene_) × 100

where A_β-carotene after 2 h_ is the absorbance value of β-carotene after 2 h of incubation and A_initial β-carotene_ is the absorbance value of β-carotene before incubation. All results were presented as the mean ± standard deviation (SD) of three replicates.

#### 2.2.7. Phenolic Composition by HPLC-DAD

The phenolic composition was assessed by high performance liquid chromatography (HPLC)-diode-array detector (DAD) [24], using a Gilson HPLC (Villers-le-bel, France) and a Finnigan/Surveyor DAD (Thermo Electron, San Jose, CA, USA). The eluent consisted of water with 0.1% trifluoroacetic acid (solvent A) and acetonitrile with 1% trifluoroacetic acid (solvent B) with a gradient starting with 0% solvent B, at a flow rate of 1 mL/min, 0% solvent B at 5 min, 20% solvent B at 15 min, 50% B at 30 min, 100% solvent B at 45 min, 100% solvent B at 50 min, 0% solvent B at 55 min and 0% solvent B at 60 min. The extract was injected into the HPLC (10 μL) and the compounds were separated on a C18 column (250 × 4.6 mm, 5 μm) (ACE, Aberdeen, Scotland). The chromatograms were recorded at wavelengths ranging from 200 to 520 nm. Phenolics were identified by their peak retention times, UV spectra and UV max absorbance bands in comparison with commercial standards (purchased from Extrasynthese, Genay, France) and data reported in the literature. Phenolics were quantified using the internal standard method, and the results expressed in mg/100 g DW as the mean ± standard deviation (SD) of three replicates.

### 2.3. Antimicrobial Assays

#### 2.3.1. Extraction and Bacterial Strains

For each sweet cherry cultivar sample, 800 mg of dry weight (DW) was weighed and mixed with 10 mL of methanol 80%, thoroughly in a vortex. The mixtures were heated at 70 °C for 20 min, and then centrifuged at 13,000 rpm, 1 °C for 15 min (Eppendorf Centrifuge 5804 R, Hamburg, Germany). The supernatants were collected, filtered with Spartan filters (0.2 mm) and solvent removed under vacuum. The extracts were dissolved in 10% dimethylsulfoxide (DMSO) (Sigma-Aldrich, Steinheim, Germany) to a concentration of 1 mg/mL to be used in disk diffusion assay. Bacterial isolates of Gram negative (*Klebsiella pneumoniae* MJS281 and *Enterobacter aerogenes* MJH813) were collected from urine and Gram positive (*Staphylococcus aureus* MJS241 and *Enterococcus faecalis* MJS257) were collected from fecal samples from patients hospitalized in various departments of Hospital Centre of Trás-os-Montes and Alto Douro (CHTMAD, Vila Real, Portugal)—these are located in the cities of Lamego, Peso da Régua, Chaves, and Vila Real, Portuguese north province of Trás-os-Montes and Alto Douro. Ethical approval for this study was granted by the Ethics Committee of Hospital Vila Real (CHTMAD, Vila Real, Portugal), according to a research collaboration protocol established in 2003. Bacteria was isolated and identified by standard biochemical classification technique followed by genetic identification over 16S rRNA sequencing. American Type Culture Collection (ATCC) of *E. coli* and *S. aureus* were also used in the antimicrobial assays. Isolates were freshly prepared and inoculated in Petri dishes with Brain Heart Infusion (BHI) agar medium and incubated overnight at 37 °C.

#### 2.3.2. Disk Diffusion Assay

Disk diffusion assay [25] was used to screen the antibacterial effect of plant extracts. Test isolates were grown on nutrient agar plates and later inoculated on nutrient broth to log phase. Test bacterial suspensions turbidity was adjusted to 0.5 MacFarland units. Petri dishes were prepared with 20 mL of Mueller-Hinton Agar (Oxoid, UK) and seeded with bacterial suspensions. Sterile paper disks were placed on an agar plate and impregnated with 15 μL of each previous extract solution in DMSO. Plates were incubated at 37 °C for 24 h. Disks of gentamicin (CN10–Oxoid CT0024B, UK) were used as positive control and disks impregnated with DMSO were used as negative control. After incubation, the diameter (mm) of the inhibitory zone around the disk was recorded. All the tests were performed in triplicate and antibacterial activity was expressed as the mean of inhibition diameters (mm) produced. The percentage of relative inhibition zone diameter (%RIZD) of each extract was calculated, according to Aires et al. [26] by the application of the following equation:

%RIZD = ((IZD_sample_ − IZD_negative control_)/IZD_antibiotic standard_) × 100

where IZD is the inhibition zone diameter (mm).

### 2.4. Statistical Analysis

Data is presented as mean ± standard deviation of three replicates, and the results presented by dry weight (DW). Differences among means were determined by analysis of variance (ANOVA), using SPSS (Statistical Package for Social Sciences) software, version 24.0 (IBM Corporation, New York, NY, USA) software. The fulfillment of the ANOVA requirements, namely the normal distribution of the residuals and the homogeneity of variance, were evaluated by means of the Shapiro–Wilk’s test (*n* < 50), and the Levene’s tests, respectively. All dependent variables were analyzed using a one-way ANOVA with or without Welch correction, depending if the requirement of the homogeneity of variances was fulfilled. If statistically significant effect was found, comparison of means was performed using Tukey’s multiple comparison test. All statistical tests were performed at a 5% significance level. Pearson correlations and regression analysis were also calculated using SPSS, but, in the latter situation, only those presenting a R^2^ value > 0.5 are referred.

## 3. Results and Discussion

### 3.1. Total Phenolics, Flavonoids, Photosynthetic Pigments and Antioxidant Activity

#### 3.1.1. Extracts from Stems

Regarding stem’s extracts, significant differences were observed in several of the analyzed parameters, being the exception the *ortho*-diphenols and the antioxidant activity measured by the DPPH and β-carotene methods (Table 1).

The total phenolic content ranged from 32.49 mg/g in cv. *Lapins*, to 23.59 mg/g recorded in stems of cv. *Early Bigi* C. Similarly, total flavonoids presented the same trend, with higher values in cv. *Lapins* (24.75 mg/g) and lower in stems of cv. *Early Bigi* C (13.06 mg/g). Although these values are hard to compare, due to the lack of previous works, the total phenolic and flavonoids content can be considered similar to the ones reported by Bastos et al. [10]. Other reports show higher amount of total phenolics and similar content of flavonoids [27], or comparable values [28]. Saponins, a group of glycosides, known to have a bitter taste, are widely distributed in plants and known by their pharmacological properties [29] that makes of them the main constituents of many plant drugs, folk medicines and are responsible for most of the observed biological effects [30,31], were present in higher amount in stems of cv. *Lapins* (181.12 mg/g), with similar amount recorded for cv. *Early Bigi* NC (101.79 mg/g). For cvs. *Early Bigi* C and *Burlat*, the content of saponines were lower, with 42.45 and 38.05 mg/g, respectively. No previously data is available regarding saponin content in sweet cherry stems, being these the first data about the presence of those compounds in this samples. To provide same comparison values, works done with tea leaves, one of the most studied plant species regarding biochemical and bioactive properties, showed similar values of saponins content [32].

Overall, content in the photosynthetic pigments (chlorophyll a, b, and carotenoids) was found to be higher in samples of cv. *Early Bigi* NC, although with other samples showing similar amounts of Cla (*Early Bigi* C) and Clb (*Burlat* and *Early Bigi* C). The antioxidant activity only differed between cultivars for the FRAP methodology, with enhanced results when using cv. *Lapins* stems. For this methodology, results were found to correlate with the content in flavonoids (y = 0.8899x + 1.5521, R^2^ = 0.781) and saponins (y = 0.0874x + 10.085, R^2^ = 0.824). There was also a correlation between DPPH radical scavenging activity and the FRAP assay, but with low R^2^ (0.54), probably due to the fact that compounds that react with the DPPH˙ radical might not be the same as those reacting with the Fe^3+^-TPTZ complex. In the DPPH method, the radical is neutralized when receiving H^+^ and/or electrons from antioxidants, but for the FRAP assay the Fe^3+^-TPTZ complex is reduced to TPTZ-Fe^2+^ only by an electron transfer mechanism, by compounds with redox potential below 0.7 V [33]. Furthermore, the measure of reducing power appears to be related to the degree of hydroxylation and extent of conjugation in polyphenols [34], and the FRAP assay is not able to detect compounds that act by radical quenching (H^+^ transfer), particularly thiols and proteins [35]. The use of other methodologies to access the antioxidant activity of extracts could also provide more information about the underlying mechanisms and help to explain these differences among spectrophotometric methods. One approach could be the use of cyclic voltammetry (CV), an electrochemical methodology that is fast and cheap [36]. This technique is commonly employed to investigate the reduction and oxidaqtion processes of molecular species, to which good correlations between the oxidation potentials of various antioxidant and their antioxidant efficiency have been found [37]. Indeed, strong correlations have been found between DPPH, ABTS, FRAP and reducing power assays and CV [38]. However, correlations of CV to metal chelation ability and advanced glycation end products formation are weak, implying that different mechanisms are behind the antioxidant effects [39].

Significant antioxidant activity was already found for several types of extracts of cherry stems [7,8,9,27,28], with correlations recorded between phenolic and flavonoids in one or more antioxidant evaluation tests performed [7,8,9,28].

#### 3.1.2. Extracts from Kernels

For kernel’s extracts, statistically significant differences were observed in all tested parameters, with the exception of β-carotene method (Table 2).

The content of total phenolics was higher in cvs. *Early Bigi* NC (2.76 mg/g) and *Burlat* (2.46 mg/g), with the former presenting also increased content in total flavonoids and *ortho*-diphenols. These two groups of compounds were not recorded in cvs. *Lapins* and *Van*, and *Early Bigi* C, *Lapins* and *Van*, respectively. Saponin content ranged from 8.34 mg/g in cv. *Lapins* to 49.62 mg/g in cv. *Early Bigi* C, results opposed to those verified for stems, with regard to their content in both cultivars. As for stems, there is no previously data available regarding kernels saponin content, being this work the first one concerning saponins content in kernels of sweet cherry, although some works performed with plants also belonging to the genus *Prunus* (*Prunus armeniaca* L.) kernel extracts have been performed and showed similar saponin content [40].

The recorded antioxidant activity was also significantly different when comparing samples, with the exception of the β-carotene assay. For DPPH and FRAP methods, higher antioxidant activity was found for samples of cvs. *Early Bigi* NC and *Burlat*. Again, all these data are difficult to compare, as this is one of the first reports concerning this type of samples. However, phenolic content of cherry kernels has been reported earlier, ranging from 601 to 1648 µg/g [41] for cvs. *Grace Star*, *Black Star*, *Vigred* and *Vera* or as low as about 320 µg/g, for an unidentified cultivar grown in Slovenia [42]. In both works, the flavonoid group corresponds to about one third of the quantified phenolics, considerably different of the flavonoid content recorded in the present work. The antioxidant activity of sweet cherry kernels has been poorly studied, but it has been recorded previously, with about 90% of inhibition of DPPH radicals [43]. In the present work, the antioxidant activity, either using the DPPH (y = 1.7147x + 0.6952, R^2^ = 0.958) or the FRAP (y = 3.55x + 0.5148, R^2^ = 0.950) method was found to correlate positively with the phenolic content of samples. There was also a strong correlation between DPPH and FRAP methods, which can indicate that the compounds responsible for the antioxidant activity are the same [28]. This is further confirmed by the positive correlations also found for individual compounds. The results for the DPPH method were also found to correlate strongly with values of *ortho*-diphenols (y = 5.314 + 0.4090, R^2^ = 0.8674) and flavonoid content (y = 0.3257x + 0.3754, R^2^ = 0.9079).

### 3.2. Identification of Phenolic Compounds

#### 3.2.1. Extracts from Stems

**Figure 2:**
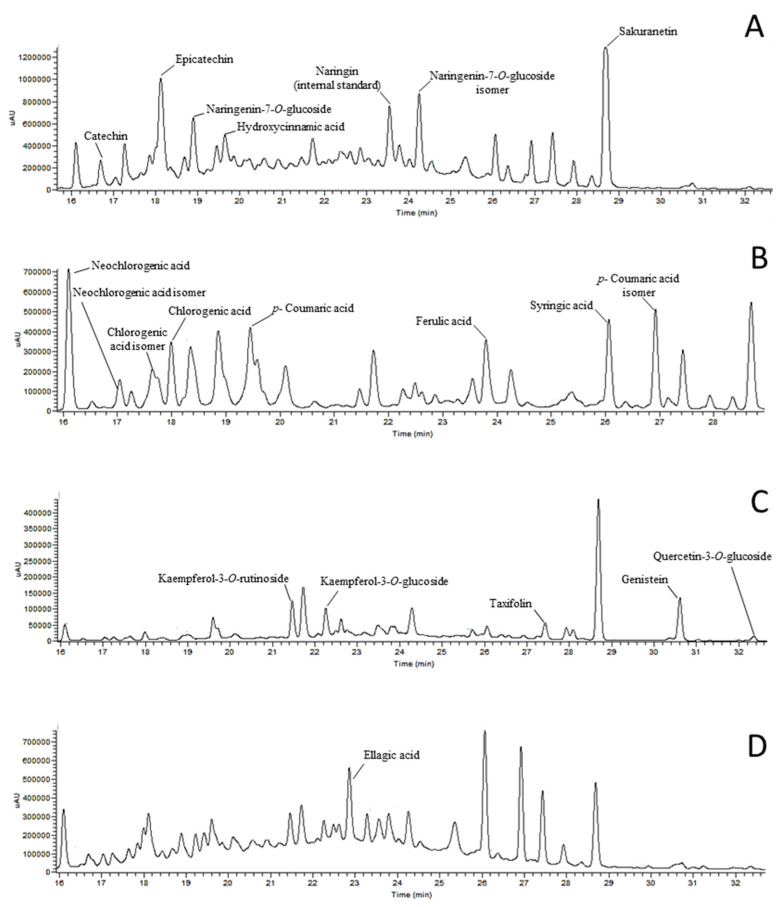
Example of a HPLC chromatograms of phenols detected in sweet cherry stem’s extracts, recorded at different wavelengths: 280 nm (**A**), 320 nm (**B**), 370 nm (**C**) and 254 nm (**D**).

The phenolic profile of sweet cherry stem’s extracts is showed in Table 3 and Figure 2.

**Figure 3:**
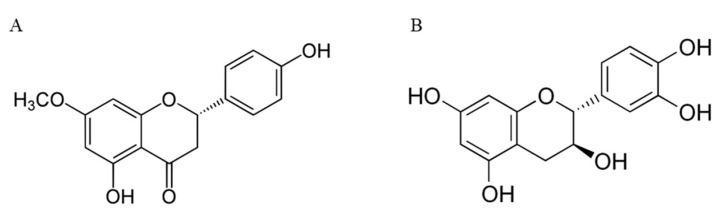
Chemical structure of sakuranetin (**A**) and catechin (**B**).

The total amount of phenolic compounds ranged from 434.02 mg/100 g to 589.99 mg/100 g in cvs. *Van* and *Early Bigi* NC respectively, and 16 individual compounds were identified in extracts of stems. The content of each phenolic compound varied significantly when comparing cultivars, with only syringic acid present in similar amounts in all cultivars. In all samples, the most abundant compound was sakuranetin (Figure 3), with the exception of stems of cv. *Early Bigi* NC, where the most abundant compound was the ellagic acid. Of the identified compounds, the majority have already been described to be present in cherry stems [9,10]. However, there are some differences in the number and identification of the compounds with those authors, since in the present work epicatechin and hydroxycinnamic, ellagic, chlorogenic, and syringic acids are reported and absent in the work of Bastos et al. [10]. Some works have recorded a strong relationship between the content of some phenolics present in cherry stems and antioxidant activity [9]. In the present work, only the amount of neochlorogenic acid and its isomer was strongly and positively correlated with the recorded antioxidant activity in the FRAP method (Y = 0.600x + 0.789, R^2^ = 0.879). Neochlorogenic acid, as well as other phenolic compounds, show their antioxidant properties by chelating metal ions, inhibiting radical forming enzymes and lipid oxidation, eliminating free radicals, diminishing oxidative stress levels, and thus protecting cell membranes against damage [3,8].

#### 3.2.2. Extracts from kernels

**Figure 4:**
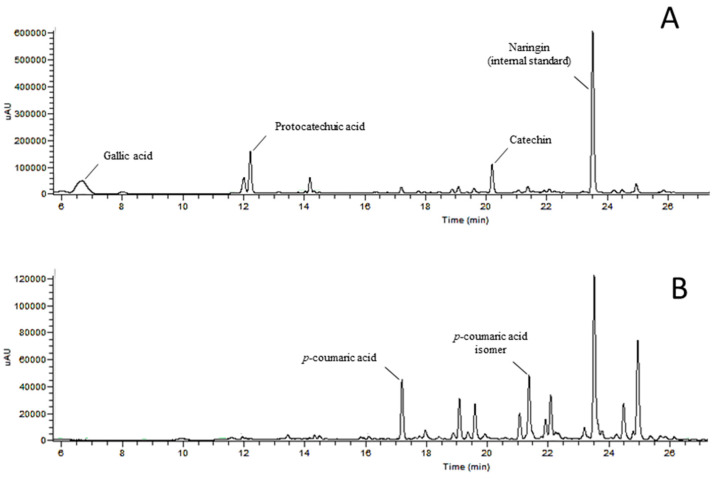
Example of a HPLC chromatograms of phenols detected in sweet cherry kernel’s extracts, recorded at different wavelengths: 280 nm (**A**) and 320 nm (**B**).

Regarding kernel’s extracts, only four compounds were found in the present work (Table 4 and Figure 4).

The total amount of phenolic compounds in kernel’s extracts ranged from 16.58 mg/100 g to 75.58 mg/100 g in cvs. *Van* and *Ealy Bigi* NC, respectively. The most abundant phenolic compound was catechin (Figure 3), in four of the five samples, with protocatechuic acid being the major one in kernels of cv. *Early Bigi* NC, again showing the effect of tree cover in chemical parameters of sweet cherries. Previous works have reported the presence of anthocyanins, but also epicatechin, procyanidins, and flavonol glycosides [11]. Similarly, Senica et al. [41] have also identified hydroxycinnamic and hydroxybenzoic acids, including catechin and *ρ*-cumaric acid. Variations on the number and quantity of compounds can be related to extractions and analysis methodologies, as well as genotypic diversity, as previously reported for another *Prunus* [44]. Interestingly, for kernels, all identified compounds strongly correlate with the recorded antioxidant activity, namely for FRAP and DPPH, with R^2^, for all correlations, above 0.81. For the β-carotene assay, although positive correlations were found, R^2^ was always below 0.5.

### 3.3. Antimicrobial Assays

The antimicrobial activity expressed as% RIZD is showed in Table 5. The negative control (DMSO) used for the extract’s preparation showed no effectiveness against the target Gram positive and Gram negative bacteria, as expected. Extracts from kernels were not able to inhibit the growth of any of the bacterial stains tested, and their use resulted in only a small synergistic effect in three situations. By the other hand, extracts from cherry stems proved to be more effective, namely for Gram positive bacteria.

**Figure 5:**
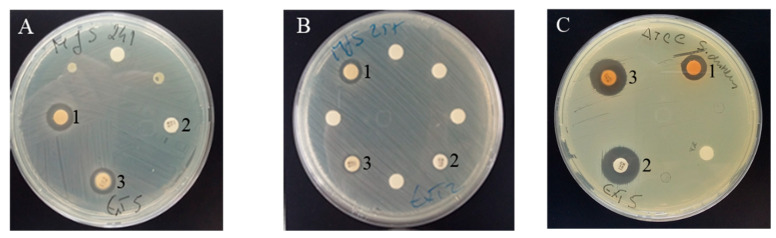
Clinical isolates of *S. aureus* MJS241 (**A**), *E. faecalis* MJS257 (**B**) and *S. aureus* ATCC (**C**) and the inhibitory effect of sweet cherry stem’s extracts. (**1**) Sweet cherry stem’s extract; (**2**) tested antibiotic (Gentamicin); (**3**) sweet cherry stem’s extract and tested antibiotic.

Indeed, for *S. aureus* ATCC, all samples were able to inhibit its growth, with values of% RIZD above 55%. More interestingly, for the clinical isolates of *S. aureus* MJS241 and *E. faecalis* MJS257, all the extracts of stems were able to inhibit the growth of those bacteria that proved to be resistant to the tested antibiotic (Figure 5). Although no significant correlation was found, this ability to inhibit bacterial growth may be due to the presence of sakuranetin, since it was the most abundant phenolic compound in all samples, although cv. *Early Bigi* NC has it in lower amount. Sakuranetin antibacterial activities have been demonstrated by several studies and subject of reviews [45]. These authors indicate that antibacterial activity might have multiple cellular targets, namely form complex with proteins through nonspecific forces such as hydrogen bonding and hydrophobic effects, as well as by covalent bond formation, inactivating microbial adhesins, enzymes, cell envelope transport proteins, among others, or even disrupting microbial membranes. Extracts of cv. *Lapins* exhibited the strongest growth suppression for the Gram positive *S. aureus* MJS241 and *E. faecalis* MJS257 (12.11 and 11.08 mm, respectively). Extracts of cv. *Burlat* presented 10.21 mm of inhibition for *S. aureus* MJS241 and 9.00 mm for *E. faecalis* MJS257. Growth inhibition of *S. aureus* MJS241 was also caused by stem extracts of cv. *Van* (10.13 mm), while extracts of cv. *Early Bigi* C showed an inhibitory effect on *E. faecalis* MJS257 (10.03 mm). Antimicrobial effect of cherry stems has already been recorded that has been linked to the content of phenolic compounds [9,27]. Indeed, in the present work, the higher values of total phenolics were found in stems of cv. *Lapins* that presented the higher% RIZD. The stems of this cultivar had the higher content in neochlorogenic acid that might be the reason of the increased antimicrobial activity. This compound presents aromatic hydroxyl groups that have high affinity to bacterial membranes, and are able to interfere with both membrane and cytoplasmic organelles, leading to the bacteria death and it recorded antimicrobial effect [46,47,48]. Neochlorogenic acid is also known to have activity against Gram positive *E. faecalis* and *S. aureus* [47].

For the tested Gram negative bacteria, no inhibitory effect was found, although extracts of cvs. *Early Bigi* C and NC and *Lapins* potentiated the antimicrobial effect of the latter when used in combination. The resistance of Gram negative bacteria is linked to the composition of cell wall components (lipopolysaccharides) that makes them less susceptible to the inhibitory effects of some phenolic compounds [49]. The analysis of correlation between the antimicrobial activity and the concentration of the phytochemical profile of sweet cherry stem’s extracts allowed to establish a significant positive correlation between the activity against *S. aureus* MJS241 and their content in neochlorogenic acid (Y = 0.16x + 5.253, R^2^ = 0.887, *ρ* < 0.001). Lou et al. [47], Nunes et al. [50] and Taofiq et al. [51] showed the strong antimicrobial activity linked to this polyphenol. A positive correlation was also recorded between the content in saponins and the inhibition of its growth rate (Y= 0.0187x + 8.1595, R^2^ = 0.6093, *ρ* < 0.001). Antimicrobial activity of saponins extracted from diverse plants has been already described by several authors (reviewed by [52]).

## 4. Conclusions

Sweet cherry is one of the most popular fruits, highly appreciated by consumers, not only for its fresh consumption but also as a processed fruit, which leads to an increase on its production. In parallel, the amount of sweet cherry by-products also increases, and the need to their valorization becomes even more important. In the present work, stems and kernels of four different cultivars of sweet cherry were characterized, providing useful information in order to find new alternative uses for such by-products. For the first time, saponins, known by their pharmacological properties, were quantified in both stems and kernels. Out of all the tested cultivars, and regarding stem’s extracts, cv. *Lapins* had overall highest bioactive content, saponins and antioxidant activity, while concerning kernel’s extracts, *Early Bigi* NC was the one with higher bioactive content and antioxidant activity. Stem’s extracts had the biggest polyphenolic compound diversity, with 16 different phenolic compounds identified. The compound present in highest amount was sakuranetin in all cultivars with exception of cv. *Early Bigi* NC, where ellagic acid was the most abundant. Only the amount of neochlorogenic acid and its isomer found in extracts from stems was strongly and positively correlated with the antioxidant activity. Four compounds were identified in sweet cherry kernel’s extracts, being catechin the most abundant. These extracts were not able to inhibit the growth of the bacterial stains tested, contrary to the observed for stem’s extracts that proved to be effective against Gram positive bacteria, namely for *S. aureus* MJS241 and *E. faecalis* MJS257 that were resistant to the tested antibiotic. The most promising extracts were those from cv. *Lapins*, as they presented increased% RIZD, probably linked to the higher amount of neochlorogenic acid, known for its recognized antibacterial activity.

This work shows that sweet cherry by-products have very interesting bioactive properties, mainly regarding to the content in phenolics, saponins, antioxidant and antimicrobial activities However, these by-products should be further explored and more studies are required, in order to explore nutraceutical and pharmacological formulations or antioxidant preservatives for the food industry and their effects on human health. Sweet cherry stem’s extracts may provide valuable solutions to the global problem of antibiotic resistance due to their antimicrobial activity linked to this unvalued by-product, although more research should be performed to test its applicability.

## Figures and Tables

**Table 1:** Bioactive content and antioxidant activity of sweet cherry stems from four different cultivars.

Cultivar	Total Phenolic (mg GAE/g)	Total Flavonoid (mg CE/g)	*Ortho*-Diphenols (mg CAE/g)	Saponins (mg DE/g)	Cla (µg/mL)	Clb (µg/mL)	Total Carotenoids (µg/mL)	DPPH (µg Trolox/g)	FRAP (µg Trolox/g)	β-Carotene (% Inhibition)
*Burlat*	26.63 ± 1.65ab	14.67 ± 3.23b	3.75 ± 0.85	38.05 ± 3.94c	3.70 ± 0.44b	2.61 ± 0.25ab	1.25 ± 0.05b	26.28 ± 1.15	15.15 ± 1.40b	90.13 ± 1.26
*Early Bigi* C	23.59 ± 0.14b	13.06 ± 1.97b	3.88 ± 0.15	42.45 ± 2.97c	4.18 ± 0.42a	2.60 ± 0.10ab	1.42 ± 0.16b	25.98 ± 1.15	12.66 ± 2.48b	88.37 ± 0.94
*Early Bigi* NC	31.30 ± 2.15a	19.71 ± 3.43ab	5.15 ± 0.97	101.79 ± 8.35a	4.91 ± 0.34a	2.89 ± 0.18a	1.58 ± 0.11a	27.48 ± 0.60	18.15 ± 2.24b	90.36 ± 4.17
*Lapins*	32.49 ± 5.23a	24.75 ± 1.14a	5.65 ± 0.75	181.12 ± 6.92a	2.76 ± 0.08c	2.04 ± 0.02c	1.25 ± 0.06b	28.06 ± 0.13	26.66 ± 2.26a	91.82 ± 0.69
*Van*	30.56 ± 1.29ab	21.18 ± 4.75ab	4.24 ± 0.70	98.66 ± 13.44b	3.64 ± 0.16b	2.43 ± 0.09bc	1.29 ± 0.07b	27.02 ± 0.58	18.21 ± 2.19b	94.96 ± 3.23
*p value*	0.012	0.006	0.113	0.000	0.000	0.001	0.008	0.060	0.000	0.070

Abbreviations: *Early Bigi* C—grown under net cover; *Early Bigi* NC—grown without net cover. Values are means  ±  SD (*n*  =  3). Different small letters (a–c) in the same row are significantly different at *p* < 0.05 according to the analysis of variance (ANOVA) and multiple range test (Tukey’s test).

**Table 2:** Bioactive content and antioxidant activity of sweet cherry kernels from four different cultivars.

Cultivar	Total Phenolic (mg GAE/g)	Total Flavonoid (mg CE/g)	*Ortho*-Diphenols (mg CAE/g)	Saponins (mg DE/g)	DPPH (µg Trolox/g)	FRAP (µg Trolox/g)	β-Carotene (% Inhibition)
*urlat*	2.46 ± 0.09a	1.81 ± 0.18b	0.09 ± 0.01b	41.83 ± 0.46b	1.05 ± 0.06a	0.58 ± 0.01a	93.46 ± 1.12
*Early Bigi* C	1.60 ± 0.19b	0.23 ± 0.03c	Nd	49.62 ± 1.33a	0.56 ± 0.06b	0.32 ± 0.02b	89.66 ± 3.30
*Early Bigi* NC	2.76 ± 0.14a	2.59 ± 0.44a	0.16 ± 0.02a	23.53 ± 6.05c	1.17 ± 0.06a	0.59 ± 0.04a	93.95 ± 0.47
*Lapins*	1.29 ± 0.09bc	Nd	Nd	8.34 ± 0.76d	0.30 ± 0.08c	0.20 ± 0.03c	89.13 ± 2.25
*Van*	1.17 ± 0.13c	Nd	Nd	13.18 ± 1.27d	0.30 ± 0.01c	0.19 ± 0.01c	91.31 ± 0.50
*p value*	0.000	0.000	0.000	0.000	0.000	0.000	0.062

Abbreviations: *Early Bigi* C—grown under net cover; *Early Bigi* NC—grown without net cover. Nd—not detected. Values are means  ± SD (*n*  =  3). Different small letters (a–d) in the same row are significantly different at *p* < 0.05 according to the analysis of variance (ANOVA) and multiple range test (Tukey’s test).

**Table 3:** Phenolic composition of sweet cherry stems (mg/100 g DW).

	*Early Bigi* C	*Early Bigi* NC	*Lapins*	*Burlat*	*Van*	*p Value*
Catechin	15.02 ± 2.12b	22.26 ± 0.51a	17.45 ± 0.53b	15.64 ± 0.71b	17.86 ± 0.31b	0.000
Epicatechin	59.05 ± 4.19b	74.88 ± 5.05a	50.23 ± 0.99c	61.82 ± 0.72b	49.39 ± 1.63c	0.000
Naringenin-7-*O*-glucoside	22.78 ± 2.42bc	27.52 ± 1.11a	26.61 ± 0.40ab	26.37 ± 0.44ab	20.77 ± 1.75c	0.000
Hydroxycinnamic acid	18.16 ± 3.89a	19.14 ± 0.20a	8.51 ± 0.08b	18.09 ± 0.75a	9.52 ± 0.40b	0.000
Sakuranetin	139.73 ± 11.06ab	128.31 ± 7.79b	140.88 ± 0.58ab	158.30 ± 10.08a	131.63 ± 0.85b	0.006
Ellagic acid	106.87 ± 14.56b	145.25 ± 4.69a	96.37 ± 5.59b	106.42 ± 3.93b	95.59 ± 5.26b	0.000
Neochlorogenic acid + isomer	19.11 ± 2.09d	25.63 ± 0.28c	40.62 ± 0.20a	26.38 ± 0.79c	33.11 ± 1.25b	0.000
Chlorogenic acid + isomer	43.29 ± 4.90b	51.13 ± 0.57a	23.77 ± 0.41c	19.18 ± 0.45c	25.23 ± 1.69c	0.000
*p*-Coumaric acid + isomer	18.81 ± 2.58bc	22.47 ± 0.71ab	18.38 ± 0.69c	22.56 ± 0.85a	13.46 ± 1.02c	0.000
Ferulic acid	14.95 ± 1.82a	14.28 ± 0.07a	9.56 ± 0.25bc	10.99 ± 0.57b	7.59 ± 0.65c	0.000
Syringic acid	12.63 ± 1.38	13.89 ± 0.22	9.48 ± 0.29	7.37 ± 0.33	9.15 ± 0.17	0.470
Taxifolin	7.07 ± 1.00a	8.34 ± 0.32a	4.94 ± 0.29b	4.09 ± 0.30b	4.85 ± 0.18b	0.000
Kaempferol-3-*O*-rutinoside	5.94 ± 0.60c	11.69 ± 0.20a	7.60 ± 0.17b	12.92 ± 0.69a	6.28 ± 0.67bc	0.000
Kaempferol-3-*O*-glucoside	3.20 ± 0.41c	5.95 ± 0.07a	Nd	4.29 ± 0.22b	2.70 ± 0.28c	0.000
Genistein	4.93 ± 0.74b	6.63 ± 0.13a	4.97 ± 0.08b	1.53 ± 0.07d	3.25 ± 0.09c	0.000
Quercetin-3-*O*-glucoside	16.17 ± 1.50a	8.61 ± 0.31b	Nd	3.78 ± 0.18c	0.65 ± 0.12d	0.000
Total	508.57 ± 46.57ab	589.99 ± 21.20a	493.02 ± 51.30b	501.71 ± 19.35ab	434.02 ± 9.74b	0.003

Abbreviations: *Early Bigi* C—grown under net cover; *Early Bigi* NC—grown without net cover. Nd—not detected. Values are means  ±  SD (*n*  =  3). Different small letters (a–d) in the same row are significantly different at *p* < 0.05 according to the analysis of variance (ANOVA) and multiple range test (Tukey’s test).

**Table 4:** Phenolic composition of sweet cherry kernels (mg/100 g DW).

	*Early Bigi* C	*Early Bigi* NC	*Lapins*	*Burlat*	*Van*	*p Value*
Gallic acid	4.05 ± 0.27c	13.19 ± 0.78a	3.12 ± 0.10cd	8.27 ± 0.55b	2.74 ± 0.04d	0.000
Protocatechuic acid	6.17 ± 0.41c	30.34 ± 0.86a	5.92 ± 0.14c	21.05 ± 0.42b	4.92 ± 0.06c	0.000
Catechin	10.32 ± 0.41c	20.06 ± 0.55b	6.66 ± 0.15d	22.87 ± 1.39a	7.06 ± 0.15d	0.000
*p*-coumaric acid + isomer	4.24 ± 0.15c	11.99 ± 0.32a	1.26 ± 0.05d	10.27 ± 0.48b	1.87 ± 0.31d	0.000
Total	24.78 ± 1.18c	75.58 ± 2.49a	16.97 ± 0.35d	62.46 ± 2.73b	16.58 ± 0.40d	0.000

Abbreviations: *Early Bigi* C—grown under net cover; *Early Bigi* NC—grown without net cover. Values are means  ±  SD (*n*  =  3). Different small letters (a–d) in the same row are significantly different at *p* < 0.05 according to the analysis of variance (ANOVA) and multiple range test (Tukey’s test).

**Table 5:** Antimicrobial activity (% RIZD) of cherry stems and kernels relatively to antibiotic gentamicin.

		*Burlat*		*Early Bigi* C		*Early Bigi* NC		*Lapins*		*Van*	
Standard Bacterial Strains		Stems	Kernels	Stems	Kernels	Stems	Kernels	Stems	Kernels	Stems	Kernels
	*S. aureus* ATCC	66.6	0	58.8	0	56.3	0	68.8	0	62.5	0
Gram positive	*S. aureus* MJS241	*	0	*	0	*	0	*	0	*	0
	*E. faecalis* MJS257	*	0	*	0	*	0	*	0	*	0
	*E. coli* ATCC	0	0	0	0	0	0	0	0	0	0 #
Gram negative	*K. pneumoniae* MJH812	0	0	0 #	0 #	0 #	0 #	0 #	0	0	0
	*E. aerogenes* MJH813	0	0	0	0	0	0	0	0	0	0

Means ± SD (*n* = 3). 0—extract without effect; 0–100—extract less effective than an antibiotic; *—extract effective and antibiotic without effect; # slight synergistic effect when antibiotic used in combination with extract, with increased inhibition zone compared to the former.
